# Cupping

**Published:** 1834

**Authors:** W. A. Gillespie

**Affiliations:** Ellisville, Va.


### Art. III.—Cupping.

*Remarks on the Operation of Cupping.* By W. A. Gillespie, M. D. *of Ellisville, Va.*

The use of Cupping instruments has been known from time immemorial. They are mentioned by Hippocrates and Celsus, as well as other ancent writers. No member of the profession, at this day, can be ignorant of their use and importance; but I fear there are some who, for want of proper instruments, and manual skill in the use of them, too often omit their application, and rely on other means, in no wise as clearly indicated. In the present progressive and rapid march of medical science and improvement, morbid anatomy has revealed many of the once hidden mysteries of disease; and hence a more correct pathology and diagnosis have assigned a 'local habitation and a name' to maladies formerly considered general, or rather incomprehensible. It is to this we are to attribute the increasing importance of local blood-letting as a therapeutic agent. It is not my intention or design, in this communication, to advocate or support the doctrines of any particular systems of disease; or even to give an outline of the use of cupping as a curative means; which last I leave for those better qualified, hoping your readers may be favored, from other sources, by an essay on that subject: but it is to make a few observations on the instruments, and manual operation of cupping. Various instruments formed out of different materials, have been used at successive periods. Cucurbitulæ, or small gourds, were used at remote eras, and are still used by some; but more recently, cupping glasses have nearly superseded them. The scarcity of leeches, owing to the little attention paid to procuring, preserving, and breeding them, renders cupping still more desirable as a common means of local blood-letting, especially in country practice; the cities being supplied with leechers and cuppers by profession. The common cases of cupping instruments, now used in Virginia, are sold at very high prices, and from their construction, are liable to serious objections, particularly in country practice, from the inconvenience of carrying them about, and the difficulty and attention required in keeping them in good order, &c. A great superiority is claimed for them in consequence of the exhausting syringe adapted to the glasses; but whoever makes the experiment, will find it inferior in actual practice to the old method of exhaustion by the torch. The common scarificator, too, is objectionable, on account of the great number of lancets which it contains, and which, by so great a number of incisions, were supposed to promote a freer discharge of blood, which is by no means the case. I have repeatedly observed, that more blood can be drawn from the incisions of five or six lancets, than from a larger number; and I have frequently seen the blood flow as freely from a single incision made by a scalpel or spring lancet, as from fifteen or sixteen lancets in a common scarificator. Two or three incisions made by a sharp scalpel, razor, or lancet, across a fold of the cutis vera (held or pinched up between the finger and thumb, or by an assistant) deep enough, in common cases, to go through it, will answer as well as the best scarificator, and much better than a dull or rusty one. If the scarificator be used, great care should be constantly taken to have the lancets very smooth, sharp, and free from rust; otherwise disappointment will ensue on applying the glasses, with the expectation of obtaining a free flow of blood. With respect to exhaustion of the glasses by the torch, which is preferable to the syringe, a very convenient and effectual method, is to take small pieces of what is called in the Southern States, *fat pine*, or small slips of any kind of porous wood steeped in spirits of turpentine, and dipped in flour of sulphur and dried, varying in length to suit the depth of the glasses. The end of one of these may be lighted with a candle, and the other end being held between the thumb and fore finger of the left hand, should be placed on the skin about the centre of the incisions, perpendicularly to the skin, or in the intended direction of the glass, which should be applied with the other hand without loss of time, immediately over this little torch, with one edge of the glass resting on the skin until the air is nearly exhausted, when it should be entirely applied without removing the torch, which will immediately cease to burn. The fire cannot touch or burn the patient, in consequence of the torches being longer than the diameter of the glass, on the side of which it will always rest until wholly extinguished. But as glasses are frequently broken in carrying them from place to place, an excellent substitute can be made of a small cow's horn, *Corniculum*, which may be scraped and polished until perfectly diaphanous, or transparent. Several may be used, of different capacities, from 2 to 6 oz. each. The torch may be used in the same manner with these, and they answer much better than glasses, which draw in too much of the surrounding skin, and thus, by compression of the vessels, retard the bleeding. The torches may be prepared before-hand, and conveniently carried in one of the horns or glasses, together with a piece of sponge to clean the incisions. There is another way of exhausting the horn, which is practically much better than the torch, or exhausting syringe, which last may be applied to it as well as to glasses. As it is not prevalent, I believe to any extent, in the profession, and as it may be thought by some to be void of cleanliness or decency, I shall here ask the indulgence of my more fastidious brethren, and offer as an apology, that no man ought to practice physic, who is not prepared to undergo any reproach or contumely, not implicating his principles of honor or integrity, when his proceeding is designed for the good of his patient. I allude to exhausting the air by suction with the operator's mouth. A hole is to be made through the point of the cupping horn, and stopped with common bees-wax, which, being nearly of the shape of a vial cork, is to be perforated by a common pin into the cavity of the horn, which, thus prepared, is to be applied as soon as possible over the scarifications. The air is now to be exhausted by suction, and at the same time the teeth are to be clenched on the wax, so as to stop the perforation of the pin and prevent the ingress of the air. A little practice will enable the operator to regulate at his pleasure, the degree of suction required. I repeat it, that it is particularly important that the scarifications should be made with a sharp, clean instrument, and that the horn or glass should always be applied without loss of time after making them, to prevent coagulation of the blood in the incisions, which will prevent its running. A metal stop-cock can be applied to the horn, to answer the purpose of the wax and pin; or the exhausting syringe may be adapted to it as well as to the glasses; or a thin piece of gum elastic may be confined over the perforation of the horn, which will admit of exhaustion by suction with the mouth. By this the fire or torch, one cause of dread or alarm to the patient, is removed. A similar instrument, cornicula, is described by Hildanus, cent. I. obs. 80. "It is made of horn, almost in the form of a cupping glass, except at the more slender extremity there is a perforation. The wide end is laid upon emaciated parts, and a person applying his mouth to the perforation at the small extremity, by suction draws out the air. In consequence of this, the part covered rises in the hollow of the instrument, and by this means the nutritious juices are thought to be invited to the emaciated part." Hildanus relates a cure performed by this means, and gives a figure of the instrument. Tulpius, lib. III. obs. 49, gives another instance of a cure performed by this means.

The reader will readily perceive that I have been very minute, if not tedious, in some of the foregoing descriptions, but a desire to be clearly understood by all, and to profit some, must be my apology for the same. The plan of cupping with the horn here recommended, if it has not the recommendation of neatness and show, (which in most cases is not to be disregarded) has the more substantial one of superior utility. It simplifies the operation, and adapts it peculiarly to country practice, where, as a general thing, it is most needed, as the towns (and not the country) are supplied with cuppers and leechers by profession. It obviates the necessity of procuring expensive instruments for cupping, and the inconvenience of carrying them about. It excites less dread in the mind of the patient than a formidable display of numerous and complicated instruments. This last is a matter of some importance, as the cases requiring cupping are often serious, and a strong impression of fear and dread made on the mind, may give, perhaps, a fatal turn of the scale against the languishing patient. From the simplicity and little show of the instruments and plan here recommended, no such impression is likely to be made on the patient's mind.

Any person of common dexterity, may adapt horns to the use of cupping, and a short scalpel, razor, or lancet, may be used to make the incisions. And however simple this plan may appear, I speak confidently and from several years experience, when I say, that a certain amount of blood can be sooner and more easily procured in this way, than with the most complicated and expensive instruments. I have had experience with the different instruments and modes of cupping, and am clearly of opinion that with all who prefer utility and convenience, the horn must supersede them, with some it has already done so within my acquaintance, and the conviction is daily getting stronger by experience, even with the most clamorous in favor of the syringe, glasses, &c. Some have abandoned them, and now use the horn only. In making these statements I am uninfluenced by any thing but a desire to find and communicate truth to my brethren of the profession, who will, if they make the experiment, see for themselves the grounds for them. They are the results, not only of my own, but of the experience of others in this part of the country, and I hesitate not, cordially to recommend the practice to others.

*January*, 1834.

